# The impact of BMI on long-term anthropometric and metabolic outcomes in girls with idiopathic central precocious puberty treated with GnRHas

**DOI:** 10.3389/fendo.2022.1006680

**Published:** 2022-10-03

**Authors:** Patrizia Bruzzi, Lara Valeri, Marcello Sandoni, Simona Filomena Madeo, Barbara Predieri, Laura Lucaccioni, Lorenzo Iughetti

**Affiliations:** ^1^ Pediatric Unit, Department of Paediatrics, Azienda Ospedaliero-Universitaria Policlinico, Modena, Italy; ^2^ Department of Medical and Surgical Sciences of the Mothers, Children and Adults, Post Graduate School of Paediatrics, University of Modena & Reggio Emilia, Modena, Italy; ^3^ Pediatric Unit, Department of Medical and Surgical Sciences of Mothers, Children and Adults, University of Modena & Reggio Emilia, Modena, Italy

**Keywords:** central precocious puberty, child, final height, body mass index, obesity, gonadotropin-releasing hormone analogues, insulin resistance, lipids

## Abstract

**Background:**

Gonadotropin-releasing hormone analogs (GnRHas) are effective in increasing the final height of children with idiopathic central precocious puberty (ICPP). However, in previous years, some transient metabolic complications have been described during this treatment, for which there are no long-term outcome data. Our study aimed to evaluate the efficacy of GnRHas and clarify if body mass index (BMI) at diagnosis of ICPP could influence long-term outcomes.

**Methods:**

This was an observational, retrospective study that recruited a cohort of girls with ICPP. Data for anthropometric measures, fasting lipid profile, and glucose metabolism were collected at baseline [when GnRHas treatment started (T1)], at the end of the treatment (T2), and near-final height (nFH) or final height (FH) (T3). Predicted adult height (PAH) was calculated at T1 following Bayley and Pinneau’s method. Analysis was carried out using BMI standard deviation score (SDS) categories at T1 (group A, normal weight, vs. group B, overweight/obese).

**Results:**

Fifty-seven girls with ICPP who were treated with GnRHas were enrolled in the study (group A vs. group B: 33 vs. 24 patients, aged 7.86 ± 0.81 vs. 7.06 ± 1.61 years, respectively; *p* < 0.05). In the study population, nFH/FH was in line with the target height (TH) (*p* = 0.54), with a mean absolute height gain of 11.82 ± 5.35 cm compared with PAH. Even if the length of therapy was shorter (group A vs. group B: 1.84 ± 2.15 vs. 2.10 ± 0.81 years, respectively; *p* < 0.05) and the age at menarche was younger (group A vs. group B: 10.56 ± 1.01 vs. 11.44 ± 0.85 years, respectively; *p* < 0.05) in group B than in group A, the nFH/FH gain was still comparable between the two groups (*p* = 0.95). At nFH/FH, BMI SDS was still greater in group B than in group A (*p* = 0.012), despite the fact that BMI SDS significantly increased in group A only (*p* < 0.05). Glucose metabolism got worst during GnRHa with a complete restoring after it, independently from pre-treatment BMI. The ratio of low-density to high-density lipoprotein cholesterol transiently deteriorated during treatment with GnRHas in group A only (*p* = 0.030).

**Conclusions:**

Our results confirm the effectiveness of treatment with GnRHas on growth and do not support the concern that being overweight and obese can impair the long-term outcomes of GnRHas therapy. However, the observed transient impairment of metabolic parameters during treatment suggests that clinicians should encourage ICPP girls treated with GnRHas to have a healthy lifestyle, regardless of their pretreatment BMI.

## 1 Introduction

In previous decades, the increase in childhood overweight and obesity has been considered to be a potential driving factor in the trend towards earlier age at pubertal onset (1). Several studies have reported an increased risk of anticipated or precocious puberty in girls with excess adiposity (2–5); however, the association between idiopathic central precocious puberty (ICPP) and body mass index (BMI) remains controversial, and most of the pathogenic mechanisms need to be studied further (6). The mechanisms underlying puberty are still not completely understood and include genetic and epigenetic factors, energy balance, and variation in the expression of brain neurotransmitters and neuropeptides (7, 8). The link between obesity and puberty modulates this complex net, comprising interactions of environment and genetic factors (9).

Recent data from preclinical models strongly suggest that changes in pubertal timing induced by early overfeeding are primarily attributable to perturbations of hypothalamic pathways. First, leptin, which is usually elevated in the obese, secreted by adipocytes serves as a metabolic signal for pubertal progress and appears to be a permissive factor for the onset of puberty (9, 10). Furthermore, the increased hypothalamic expression of kisspeptin, or its coding gene *Kiss1*, in overfed animals has been linked to precocious pubertal modifications (11). Recently, Heras et al. (12) reported data from animal studies that support the hypothesis that the link between obesity and early puberty is also driven by a brain pathway that involves enhanced ceramide synthesis in the hypothalamic paraventricular nucleus, which promotes accelerated pubertal maturation *via* sympathetic innervation of the ovary. In addition, insulin-like growth factor 1 (IGF-1) and glucose have been shown to be involved in the control of the secretion of gonadotropin-releasing hormone (GnRH). Furthermore, in peripheral tissues, the presence of a condition of insulin resistance characterized by high levels of insulin, commonly associated with obesity, can stimulate sex steroid production by acting on the adrenal glands and gonads, and can decrease the levels of sex-hormone-binding globulin, which results in an increase in the bioavailability of sex steroid (13).

A high BMI seems to affect not only the timing of pubertal maturation but also the pathways through which pubertal maturation takes place; in overweight and obese girls, breast development seems to appear before pubic hair development as the first manifestation of puberty (14). This pattern is different from that among normal-weight peers, and it raises the issue of the relationship of aromatase activity, adiposity, and the production of estrogens (15). Aromatase, located in the adipocyte, converts androgens to estrogens; therefore, obese girls could be exposed to a high dose of estrogens even before adrenarche.

Since 1981, gonadotropin-releasing hormone analogues (GnRHas) have been the standard in ICPP treatment (16, 17). GnRHas binds to GnRH receptors, and this action causes the desensitization of pituitary gonadotroph and the subsequent suppression of gonadal steroid secretion, resulting in the slowdown of pubertal development. In ICPP, clinicians proposed the treatment to prevent potential psychological problems related to early pubertal development and to increase final height, which was compromised by sex hormone-driven premature closure of bone growth plates (16). In this report, we aim to change the perspective and to understand if BMI at the diagnosis of ICPP could impair *per se* the treatment outcomes. Our hypothesis is that, given that overweight and obese patients usually have accelerated bone age, a faster rate of pubertal progression, and a reduced pubertal growth spurt (4), a high pretreatment BMI may reduce the effectiveness of GnRHas on increasing final height and may promote the persistence of the negative metabolic pattern or a further deterioration in the metabolic pattern in ICPP patients. Therefore, we retrospectively collected anthropometric and metabolic data from the beginning of treatment with GnRHas to near-final height (nFH) or final height (FH) among a cohort of girls with a diagnosis of ICPP who were grouped based on their pretreatment BMI.

## 2 Materials and methods

This retrospective cohort study enrolled girls with ICPP who were treated with GnRHas at our tertiary Paediatric Endocrine Clinic from January 2000 to December 2021, and who had reached FH or nFH. Patients affected by other endocrine diseases (e.g., growth hormone deficiency, congenital adrenal hyperplasia, hypothyroidism, type 1 diabetes mellitus, or familial hypercholesterolemia), organic brain lesion, or systemic diseases, and patients taking drugs interfering with height growth, body weight, and lipid and/or glucose metabolism (e.g., corticosteroids, growth hormone therapy, or chemotherapies), were excluded.

The diagnosis of ICPP was made in accordance with standard criteria, including breast development before 8 years of chronological age (CA); accelerated growth velocity (GV) after at least 6 months of observation; laboratory investigation, including GnRH stimulation test and serum estradiol levels suggesting pubertal activation; advanced bone age (BA), determined through radiography of the non-dominant hand and wrist; and normal magnetic resonance imaging (MRI) of the hypothalamus and pituitary gland (16, 18, 19).

All anthropometric and laboratory data were collected at diagnosis of ICCP [baseline, i.e., time 1 (T1)], at which point GnRHas treatment commenced, at the end of GnRHas treatment [time 2 (T2)], and at FH or nFH [time 3 (T3)], and these data were anonymously recorded in a database using alphanumeric and progressive identification codes. The discontinuation of GnRHas treatment (T2) was proposed when BA was near 12 years and/or GV slowed down to maximize the anthropometric outcomes and/or to synchronize the puberty process with peers (20).

The study was conducted in accordance with the Declaration of Helsinki and was approved by the Ethical Committee of Modena (protocol number 272/13). Parental written informed consent and patient assent were obtained at recruitment and before starting data collection.

### 2.1 Anthropometric measures

All recruited patients underwent a complete physical examination, including anthropometric measurements that were taken at each study time (T1, T2, and T3) by the same team of fully trained examiners following the Anthropometric Standardization Manual (21). Parents’ height was collected at T1, and the target height (TH) was calculated as the mid-parental height adjusted for sex (minus 6.5 cm) (22). Height (H) was measured to the nearest 0.1 cm with a calibrated wall-mounted Harpenden’s stadiometer (Holtain Ltd, Crymych, UK), was compared with age-matched reference values, and was expressed as a standard deviation score (SDS) (23). Predicted adult height (PAH) was calculated at baseline following the method of Bayley and Pinneau (24). nFH was reached after menarche, when GV was less than 2.5 cm per year during the last 6 months and/or BA was at least 14 years. FH was determined when GV was less than 1 cm per year during the last 6 months or with a hand and wrist radiographic image showing complete epiphyseal fusion. Height gain was defined as the difference between the nFH or FH and the predicted adult height at the beginning of treatment and was expressed in cm. Body weight was measured to the nearest 0.1 kg with a calibrated scale. BMI was calculated by dividing weight in kilograms (kg) by height squared (m^2^) and was standardized to SDS (BMI SDS) by age using an appropriate Italian chart (23). Patients were assigned to one of two groups based on their BMI SDS at T1: normal-weight girls (group A) and overweight and obese girls (group B) (25). The pubertal staging was assessed using the Marshall and Tanner maturity scale for girls (26).

### 2.2 Biochemical analysis

Levels of fasting lipids [total cholesterol (TC), low-density lipoprotein cholesterol (LDL-C), high-density lipoprotein cholesterol (HDL-C), and triglycerides (TGs)] were measured using *in vitro* enzyme test kits on a Hitachi system (Cholesterol CHOD-PAD, HDL-C plus, Triglycerides GPO-PAP, and LDL-C plus; Roche Diagnostics GmbH, Mannheim, Germany). The ratios of LDL-C to HDL-C and TGs to HDL-C were calculated as independent markers for cardiovascular risk (27).

Insulin was measured using an electrochemiluminescence immunoassay (IMMULITE 2000; Siemens Healthcare Diagnostics, NJ, USA). Fasting glucose was measured by enzymatic absorption photometry using a cobas^®^ 8000 analyzer (via Gluco-Quant; Roche Diagnostics GmbH). Insulin resistance (IR) was defined by using the homeostatic model assessment (HOMA) index, compared with specific pediatric percentiles according to sex and pubertal stage (28), and defined by a fasting glucose-to-insulin ratio (FGIR) of less than 7 (29).

Measurements of luteinizing hormone (LH), follicle-stimulating hormone (FSH), and estradiol (E2) were taken using the ADVIA Centaur^®^ platform (Siemens Healthcare Diagnostics, NJ, USA) using the chemiluminescent immunometric method.

All biochemical analyses were carried out in the same laboratory.

### 2.3 Diagnostic imaging

Skeletal maturation was assessed based on roentgenograms of the non-dominant hand and wrist, and BA was determined following the method of Pyle and Greulich (30) by the same team of pediatric endocrine specialists. MRI of the hypothalamus and pituitary gland was carried out in all patients.

### 2.4 Statistical analysis

Data were checked for normal distribution using the Kolmogorov–Smirnov test. Continuous data are reported as mean ± standard deviation (SD) and median and interquartile range (IQR), and categorical data as percentages. Between-group comparisons were performed using Mann–Whitney *U*-tests (for numerical variables) and Pearson’s chi-squared tests (for percentages). Within-group, variables were compared using Wilcoxon matched pairs tests. The Friedman ANOVA test was used for longitudinal comparison of variables recorded at T1, T2, and T3 in the total population and in each group. Spearman correlation was used to identify the association between anthropometric variables. For each test, statistical significance was a *p*-value < 0.05. The statistical analysis was carried out using Statistica™ software (StatSoft Inc., Tulsa, OK, USA).

## 3 Results

The medical records of 110 girls diagnosed with ICPP were reviewed retrospectively. Fifty-seven girls reached nFH or FH and, therefore, comprised our study population. In total, 42% of girls (*n* = 24/57) were classified as being overweight (*n* = 21, 87%) or obese (*n* = 3, 13%) (group B) at T1. **Table 1** showed that, at baseline, when GnRHas treatment commenced, overweight and obese girls were younger and taller than normal-weight girls (group A). Nevertheless, their H SDS did not differ from the patients in group A, adjusting for TH and BA. Clinical pubertal stage was advanced (i.e., Tanner breast stage ≥ 3) in 45% of overweight and obese girls and 37% of normal-weight girls (*p* = 0.471), and no significant differences were documented in gonadotropin levels (both basal and after stimulation) and basal estrogen levels between groups (**Table 1**).

**Table 1:** Baseline features in the total population, group A and group B (T1).

	Total population (*n* = 57)		Group A: normal weight (*n* = 33)		Group B: overweight and obese (*n* = 24)		*p*-value
Parameters	Mean ± SDS	Median (IQR, 25th–75th percentile)	Mean ± SDS	Median (IQR, 25th–75th percentile)	Mean ± SDS	Median (IQR, 25th–75th percentile)	
CA (years)	7.67 ± 1.10	7.82 (7.36–8.21)	7.86 ± 0.81	7.97 (7.39–8.47)	7.06 ± 1.61	7.47 (7.11–7.76)	0.003*
BA (years)	9.52 ± 1.45	10.00 (9.00–10.50)	9.58 ± 1.28	10.00 (9.00–10.50)	9.33 ± 1.93	9.75 (9.00–11.00)	0.984
ΔBA – CA	1.86 ± 1.09	1.95 (1.39–2.61)	1.73 ± 1.04	1.85 (1.37–2.44)	2.26 ± 1.20	2.47 (1.64–3.21)	0.101
TH (cm)	160.37 ± 4.76	160.00 (156.50–164.40)	160.50 ± 4.81	160.00 (156.00–164.60)	160.03 ± 4.80	159.00 (157.00–162.00)	0.709
TH SDS	–0.40 ± 0.81	–0.44 (–1.04 to 0.34)	–0.37 ± 0.81	–0.44 (–1.13 to 0.35)	–0.47 ± 0.85	–0.79 (–0.96 to –0.10)	0.615
H SDS	1.02 ± 1.04	1.05 (0.47–1.67)	0.86 ± 1.02	0.84 (0.42–1.60)	1.53 ± 0.96	1.74 (0.73–2.22)	0.040*
H SDS adjusted for TH	1.53 ± 0.85	1.53 (1.00–1.82)	1.35 ± 0.65	1.34 (0.92–1.82)	1.95 ± 1.10	1.66 (1.45–2.01)	0.146
H SDS adjusted for BA	–0.81 ± 0.90	–0.75 (–1.44 to –0.32)	-0.80 ± 0.90	–0.83 (–1.40 to –0.32)	–0.86 ± 0.92	–0.63 (–1.74 to –0.32)	0.924
PAH (cm)	147.14 ± 5.80	146.73 (142.64–150.71)	146.92 ± 5.92	146.72 (142.16–150.16)	147.82 ± 5.59	146.75 (143.10–151.15)	0.693
PAH SDS	–2.44 ± 0.91	–2.62 (–3.20 to –1.88)	–2.46 ± 0.92	–2.60 (–3.20 to –1.88)	–2.38 ± 0.92	–2.68 (–3.00 to –1.88)	0.879
BMI SDS	0.35 ± 0.90	0.47 (–0.42 to 0.96)	–0.01 ± 0.69	0.18 (–0.68 to 0.56)	1.48 ± 0.36	1.42 (1.21–1.87)	0.000*
GV SDS	2.46 ± 2.41	2.40 (0.47–3.90)	2.22 ± 2.31	2.35 (0.72–3.17)	3.35 ± 2.86	4.56 (0.47–4.98)	0.433
Tanner breast stage ≥ 3 (%)	40	NA	37	NA	45	NA	0.471
TC (mg/dl)	154.52 ± 25.29	154.50 (136.00–175.00)	157.41 ± 26.90	158.00 (136.00–180.00)	146.36 ± 18.76	144.00 (136.00–151.00)	0.183
LDL-C (mg/dl)	83.90 ± 21.76	83.60 (70.00–100.00)	85.80 ± 23.35	88.00 (69.00–105.00)	78.90 ± 16.78	77.00 (71.00–92.00)	0.355
HDL-C (mg/dl)	58.41 ± 13.33	57.00 (49.00–66.00)	59.20 ± 15.15	60.00 (48.00–69.00)	56.27 ± 6.19	56.00 (53.00–61.00)	0.637
TGs (mg/dl)	66.92 ± 25.57	60.00 (48.00–87.00)	65.86 ± 27.38	59.00 (44.00–79.00)	70.00 ± 20.37	66.00 (54.00–93.00)	0.403
LDL-C-to-HDL-C ratio	1.48 ± 0.49	1.42 (1.19–1.64)	1.50 ± 0.54	1.46 (1.00–1.66)	1.41 ± 0.35	1.33 (1.24–1.52)	0.505
TG-to-HDL-C ratio	1.27 ± 0.73	1.01 (0.74–1.86)	1.25 ± 0.80	0.91 (0.68–1.88)	1.30 ± 0.50	1.14 (0.88–1.77)	0.388
FGIR	17.79 ± 10.64	15.47 (9.38–23.33)	19.92 ± 10.61	18.41 (12.09–24.72)	11.07 ± 8.05	7.97 (7.39–11.64)	0.017*
HOMA index	1.33 ± 0.88	1.02 (0.67–1.96)	1.16 ± 0.85	0.80 (0.64–1.42)	1.88 ± 0.82	1.99 (1.29–2.41)	0.039*
E2 (pg/ml)	25.40 ± 20.09	16.00 (11.00–36.00)	21.73 ± 15.08	15.00 (11.00–24.00)	36.96 ± 28.88	33.00 (12.90–53.00)	0.185
LH peak (mIU/ml)	13.71 ± 14.45	8.15 (5.30–17.20)	14.20 ± 15.06	7.95 (5.30–17.50)	11.96 ± 12.52	9.10 (5.05–11.75)	0.762
FSH peak (mIU/ml)	13.40 ± 6.25	12.05 (8.90–17.00)	13.94 ± 6.84	12.40 (8.50–17.60)	11.49 ± 2.95	11.75 (9.20–13.55)	0.441
Basal LH (mIU/ml)	0.92 ± 1.28	0.25 (0.10–1.30)	0.85 ± 1.26	0.20 (0.10–0.90)	1.18 ± 1.39	0.40 (0.10–2.10)	0.519
Basal FSH (mIU/ml)	3.29 ± 1.97	3.00 (1.90–4.50)	3.14 ± 1.67	3.00 (2.00–4.40)	3.84 ± 2.82	3.60 (1.30–6.50)	0.725

BA, bone age; BMI, body mass index; CA, chronological age; E2, estradiol; FGIR, fasting glucose-to-insulin ratio; FSH, follicle-stimulating hormone; GV, growth velocity; H, height; HDL-C, high-density lipoprotein cholesterol; HOMA, homeostatic model assessment of insulin resistance; IQR, interquartile range; LDL-C, low-density lipoprotein cholesterol; LH, luteinizing hormone; PAH, predicted adult height; PTS; patients; SDS, standard deviation score; TC, total cholesterol; TH, target height; TG(s), triglyceride(s). Data are reported as mean ± SDS and median (IQR, 25th–75th percentile). *p < 0.050 group A vs. group B using Mann–Whitney U-test (for numerical variables) and Pearson’s chi-squared test (for percentage values). Number (p value) in bold are ones with a statistical significance (p < 0.005). NA, not available.

At the end of treatment, as shown in **Table 2**, patients in group B presented a higher H SDS than normal-weight girls only when the parameter was adjusted for TH. The BMI SDS of patients in group B was consistently higher than in patients in group A. The mean length of GnRHas treatment was shorter in group B than in group A (**Table 2**).

**Table 2:** Anthropometric and biochemical features in the total population, group A and group B at the end of gonadotropin-releasing hormone analog (GnRHa) treatment (T2).

	Total population		Group A: normal weight		Group B: overweight and obese		*p*-value
Parameters	Mean ± SDS	Median (IQR, 25th–75th percentile)	Mean ± SDS	Median (IQR, 25th–75th percentile)	Mean ± SDS	Median (IQR, 25th–75th percentile)	
CA (years)	9.92 ± 0.75	9.84 (9.32–10.52)	10.10 ± 0.70	10.10 (9.67–10.60)	9.32 ± 0.61	9.23 (8.99–9.49)	0.000*
BA (years)	11.56 ± 0.56	11.50 (11.00–12.00)	11.64 ± 0.57	12.00 (11.00–12.00)	11.30 ± 0.48	11.00 (11.00–11.50)	0.062
Δ BA–CA	1.61 ± 0.98	1.66 (0.70–2.30)	1.49 ± 1.00	1.58 (0.64–2.23)	1.98 ± 0.81	2.06 (1.72–2.63)	0.119
Length of GnRHa treatment (years)	1.93 ± 0.93	1.99 (1.48–2.45)	2.10 ± 0.81	2.15 (1.50–2.46)	1.84 ± 2.15	1.48 (1.08–2.25)	0.047*
H SDS	0.90 ± 1.06	0.93 (0.42–1.69)	0.76 ± 1.06	0.71 (0.13–1.48)	1.38 ± 0.94	1.28 (0.83–2.35)	0.054
H SDS adjusted for TH	1.34 ± 0.85	1.46 (0.97–1.79)	1.15 ± 0.78	1.29 (0.85–1.56)	1.79 ± 0.86	1.86 (1.48–1.94)	0.008*
H SDS adjusted for BA	–0.59 ± 0.92	–0.65 (–1.31 to 0.08)	–0.63 ± 0.94	–0.59 (–1.40 to 0.04)	–0.47 ± 0.90	–0.79 (–1.10 to 0.26)	0.709
BMI SDS	0.55 ± 0.89	0.68 (–0.06 to 1.30)	0.28 ± 0.84	0.46 (–0.35 to 0.91)	1.45 ± 0.28	1.53 (1.34–1.87)	0.000*
GV SDS	–0.47 ± 2.02	–0.36 (–1.94 to 0.90)	–0.56 ± 1.98	–0.49 (–1.83 to 0.70)	–0.16 ± 2.17	0.17 (–2.05 to 1.18)	0.641
TC (mg/dl)	160.81 ± 32.57	154.00 (140.00–176.50)	162.91 ± 35.87	154.00 (140.00–180.00)	154.50 ± 22.69	158.50 (137.00–172.00)	0.761
LDL-C (mg/dl)	86.07 ± 20.88	91.50 (71.00–102.00)	86.50 ± 22.07	88.50 (71.00–103.00)	85.00 ± 20.63	91.50 (72.50–97.50)	0.777
HDL-C (mg/dl)	52.06 ± 10.98	54.00 (42.00–60.00)	51.09 ± 11.04	46.00 (42.00–57.00)	54.75 ± 11.95	59.50 (48.00–61.50)	0.472
TGs (mg/dl)	67.92 ± 30.64	68.00 (37.00–92.00)	69.80 ± 30.11	68.00 (49.00–91.00)	63.25 ± 36.16	64.50 (32.00–94.50)	0.943
LDL-C-to-HDL-C ratio	1.71 ± 0.57	1.66 (1.31–2.20)	1.74 ± 0.56	1.71 (1.31–2.20)	1.65 ± 0.67	1.61 (1.19–2.10)	0.943
TG-to-HDL-C ratio	1.28 ± 0.68	1.31 (0.64–1.61)	1.28 ± 0.58	1.31 (0.79–1.59)	1.29 ± 0.97	1.08 (0.51–2.07)	0.938
FGIR	9.14 ± 4.81	8.88 (5.00–11.03)	10.37 ± 4.75	10.44 (7.72–11.34)	5.06 ± 2.20	5.00 (2.89–7.29)	0.075
HOMA index	2.41 ± 1.31	2.15 (1.29–3.56)	2.10 ± 1.24	1.81 (1.25–2.30)	3.44 ± 1.16	3.56 (2.22–4.54)	0.150
E2 (pg/ml)	13.95 ± 6.56	10.00 (10.00–16.50)	13.83 ± 6.72	10.00 (10.00–17.00)	14.27 ± 6.38	11.60 (10.00–16.00)	0.443
Basal LH (mIU/ml)	0.32 ± 0.38	0.20 (0.10–0.40)	0.33 ± 0.43	0.20 (0.10–0.30)	0.29 ± 0.20	0.25 (0.10–0.40)	0.672
Basal FSH (mIU/ml)	1.21 ± 1.15	1.00 (0.70–1.42)	1.27 ± 1.31	0.90 (0.70–1.42)	1.08 ± 0.52	1.00 (0.73–1.30)	0.649

BA, bone age; BMI, body mass index; CA, chronological age; E2, estradiol; FGIR, fasting glucose-to-insulin ratio; FSH, follicle-stimulating hormone; GV, growth velocity; GnRHa, gonadotropin-releasing hormone analog; H, height; HDL-C, high-density lipoprotein cholesterol; HOMA, homeostatic model assessment of insulin resistance; IQR, interquartile range; LDL-C, low-density lipoprotein cholesterol; LH, luteinizing hormone; PAH, predicted adult height; PTS; patients; SDS, standard deviation score; TC, total cholesterol; TH, target height; TG(s), triglyceride(s). Data are reported as mean ± SDS and median (IQR, 25–75th percentile). *p < 0.050 group A vs. group B using Mann–Whitney U-test. Number (p value) in bold are ones with a statistical significance (p < 0.005).

**Figure 1:**
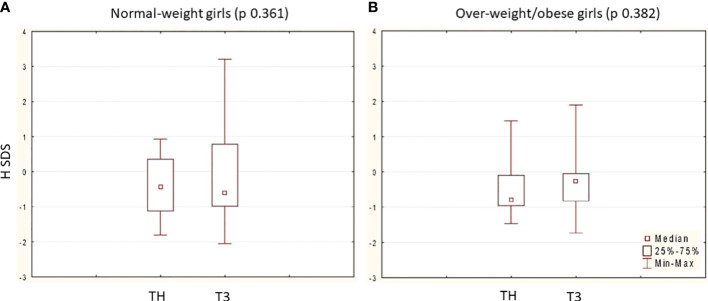
Comparison between nFH/FH SDS (T3) and TH SDS in the two group: **(A)** 33 normal-weight girls (TH SDS vs. nFH/FH SDS p 0.361) and **(B)** 24 over-weight/obese girls (TH SDS vs. nFH/FH SDS p 0.382). Data are expressed as median, 25˚ and 75˚ percentile and range (min-max). In each group, p is calculated at Wilcoxon matched pairs test.

At nFH/FH, the mean H gain was 11.82 ± 5.35 cm, and the mean nFH/FH SDS was in line with TH SDS (*p* = 0.541) without differences between groups (**Table 3**). Both normal-weight and overweight/obese girls reached an nFH/FH within their TH range (**Figure 1**). Moreover, pretreatment BMI SDS was positively associated with BMI SDS at nFH/FH (*r* = 0.55; *p* < 0,005) and inversely with age at menarche (*r =* –0.40; *p* < 0.005), but not with achieved nFH/FH SDS (*r* = –0.001) and total H gain (*r =* 0.07).

**Table 3:** Anthropometric and biochemical features in total population, groups A and B at the achievement of near-final height (nFH) or final height (FH) (T3).

	Total population		Group A: normal weight		Group B: overweight and obese		*p*-value
Parameters	Mean ± SDS	Median (IQR, 25th–75th percentile)	Mean ± SDS	Median (IQR, 25th–75th percentile)	Mean ± SDS	Median (IQR, 25th–75th percentile)
CA (years)	16.88 ± 4.13	15.59 (13.38–20.56)	16.84 ± 4.33	14.90 (13.11–20.75)	17.00 ± 3.56	16.30 (14.83–18.71)	0.522
nFH or FH (cm)	158.67 ± 6.18	157.80 (154.30–161.60)	158.53 ± 6.25	157.60 (153.90–161.60)	159.11 ± 6.15	157.90 (156.00–162.10)	0.795
nFH or FH SDS	–0.20 ± 1.11	–0.48 (–0.95 to 0.55)	–0.17 ± 1.18	–0.60 (–0.99 to 0.78)	–0.28 ± 0.91	–0.26 (–0.83 to –0.05)	0.955
nFH or FH SDS adjusted for TH	0.34 ± 0.88	0.23 (–0.41 to 1.12)	0.35 ± 0.90	0.11 (–0.33 to 1.14)	0.32 ± 0.88	0.56 (–0.42 to 0.93)	0.907
H gain (cm)	11.82 ± 5.35	12.11 (8.80–16.16)	11.92 ± 4.58	11.93 (9.69–14.63)	11.53 ± 7.52	14.29 (6.45–16.64)	0.823
H gain (SDS)	2.24 ± 0.98	2.39 (1.72–2.66)	2.33 ± 0.79	2.30 (1.72–2.66)	1.97 ± 1.44	2.47 (1.86–2.69)	0.816
BMI SDS	0.37 ± 1.12	0.47 (–0.67 to 1.05)	0.18 ± 0.99	0.21 (–0.70 to 0.69)	0.96 ± 1.33	1.03 (0.69–1.82)	0.012*
GV SDS	–3.52 ± 1.78	–3.80 (–4.50 to –3.03)	–3.76 ± 1.13	–3.80 (–4.50 to –3.03)	–2.17 ± 1.60	–2.17 (–5.14 to 1.08)	0.921
TC (mg/dl)	162.78 ± 31.16	161.00 (143.00–177.00)	165.44 ± 28.34	163.50 (147.50–183.00)	155.92 ± 37.78	150.00 (135.00–163.00)	0.091
LDL-C (mg/dl)	88.51 ± 24.46	87.00 (75.00–100.00)	90.46 ± 25.40	88.00 (78.00–109.00)	83.92 ± 22.28	83.50 (73.00–92.00)	0.328
HDL-C (mg/dl)	60.78 ± 13.06	62.00 (51.00–68.00)	61.87 ± 12.12	64.00 (51.00–69.00)	58.21 ± 15.24	57.50 (48.00–67.00)	0.235
TGs (mg/dl)	77.48 ± 32.54	72.00 (54.00–95.00)	78.61 ± 32.71	72.00 (54.00–96.00)	74.57 ± 33.13	69.50 (54.00–84.00)	0.665
LDC-to-DL-C ratio	1.49 ± 0.47	1.40 (1.19–1.76)	1.50 ± 0.49	1.40 (1.20–1.69)	1.49 ± 0.42	1.40 (1.19–1.93)	0.898
TG-to-HDL-C ratio	1.34 ± 0.71	1.18 (0.78–1.64)	1.34 ± 0.75	1.18 (0.75–1.55)	1.35 ± 0.64	1.16 (0.90–1.71)	0.771
FGIR	14.19 ± 9.13	12.25 (7.41–18.25)	14.71 ± 8.71	13.82 (7.93–17.87)	13.08 ± 10.26	11.08 (6.61–18.47)	0.492
HOMA index	1.71 ± 1.53	1.30 (0.88–1.97)	1.39 ± 0.92	1.16 (0.79–1.77)	2.39 ± 2.28	1.49 (1.04–2.29)	0.119

BMI, body mass index; CA, chronological age; FGIR, fasting glucose-to-insulin ratio; FH, final height; GV, growth velocity; H, height; HDL-C, high-density lipoprotein cholesterol; HOMA, homeostatic model assessment of insulin resistance; IQR, interquartile range; LDL-C, low-density lipoprotein cholesterol; nFH, near-final height; SDS, standard deviation score; TC, total cholesterol; TH, target height; TG(s), triglyceride(s). Data are reported as mean ± SDS and median (IQR, 25th–75th percentile). *p < 0.050 group A vs. group B using Mann–Whitney U-test. Number (p value) in bold are ones with a statistical significance (p < 0.005).

In the total population, the age of menarche was 11.22 ± 0.96 years, and it occurred 1.45 ± 1.19 years from the end of GnRHas treatment. Girls presented menarche more precociously than normal-weight girls if they were overweight and obese at T1 (group B vs. group A: 10.56 ± 1.01 vs. 11.44 ± 0.85 years, respectively; *p* = 0.008) even without any difference in time lag from the end of the treatment (group B vs. group A: 1.42 ± 1.11 vs. 1.32 ± 0.60 years, respectively; *p* = 0.501).

**Figure 2:**
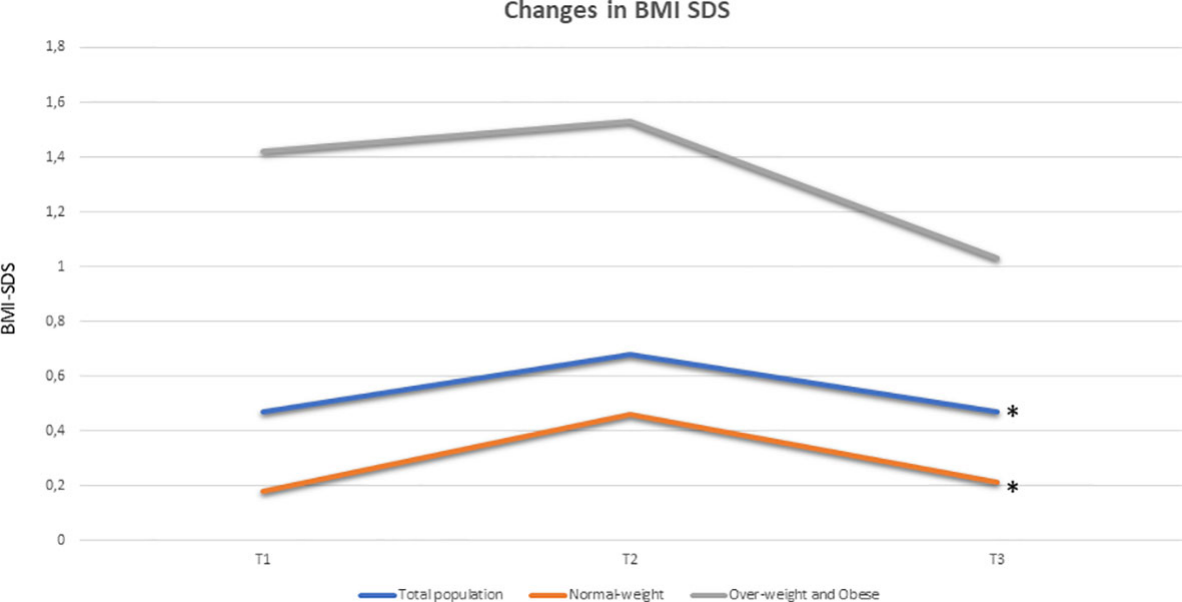
Longitudinal changes in BMI SDS (expressed as median) in total population (blue), in group A (33 pre-treatment normal-weight girls, coloured in orange) and in group B (24 pre-treatment over-weight and obese girls, coloured in grey): a significant rise in BMI SDS during GnRHa therapy was documented in the population (*χ* ^2^ 11.04, p 0.003) and the normal-weights group (*χ* ^2^ 10.07, p 0.006), while overweights and obese girls did not show any significant changes (*χ* ^2^ 3, 86, p 0.238). At T3, BMI SDS returned near to pre-treatment values both in the total population and in group A, while it seems to decrease respect to pre-treatment mean data in group B (no statistical significance). Legends: BMI SDS, Body Mass Index Standard Deviation Score; *p<0.05 at Friedman ANOVA test.

Longitudinal analysis documented a significant rise in BMI SDS during GnRHas treatment in the total population (*χ* ^2^ = 11.04; *p* = 0.003), which was attributable to the slight increase in the normal-weight group (*χ* ^2^ = 10.07; *p* = 0.006). Overweight and obese girls did not show any significant changes (*χ* ^2^ = 3.86; *p* = 0.238) (**Figure 2**). At the achievement of nFH or FH, BMI SDS returned to near pretreatment values in the total population and group A, whereas it only appeared to decrease in relation to the pretreatment mean data in group B (**Figure 2**).

At baseline, lipid values were normal in both groups, whereas glucose metabolism indexes were worse in group B than in group A, even if the mean values of FGIR and HOMA index were within the normal range in both groups (**Table 1**).

**Figure 3:**
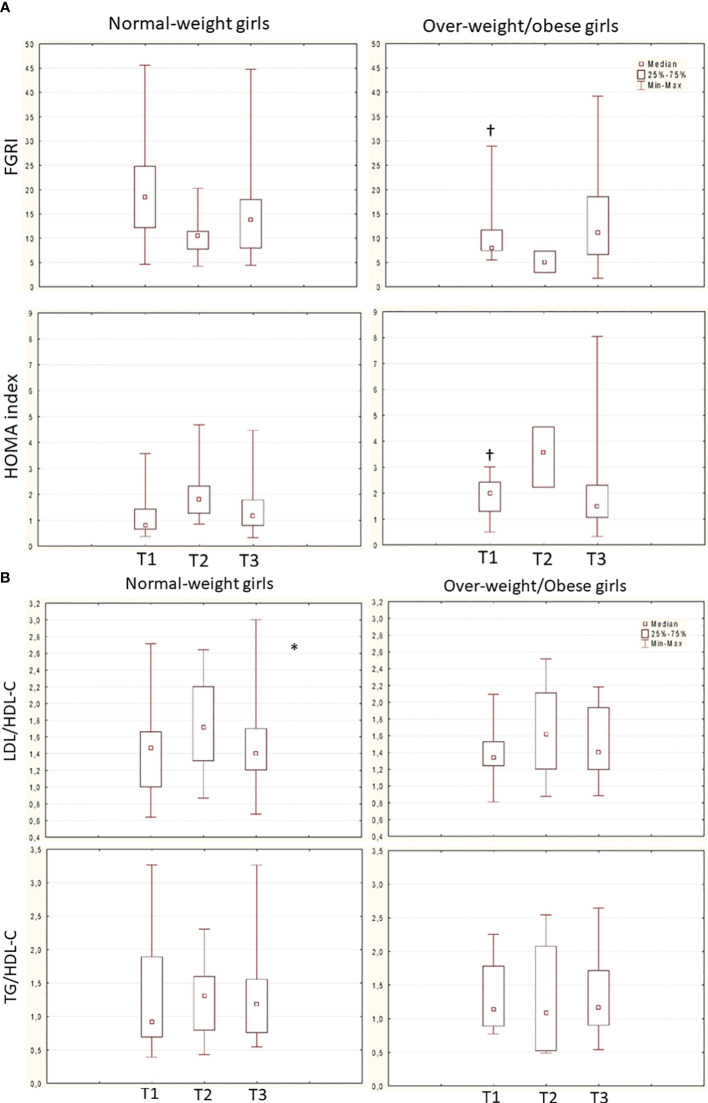
**(A)** Trend of FGRI (normal-weight: *χ* ^2^ 4.8, p 0.090; overweight/obese: *χ* ^2^ 4.66, p 0.969) and HOMA index (normal-weight: *χ* ^2^ 5.2, p 0.07; overweight/obese: *χ* ^2^ 4.66, p 0.096) over time in the 2 groups (Friedman ANOVA test). ^†^ p<0.050 Group A vs. B (mann-whitney U test). Data are expressed as median, 25°-75° percentile overlapped min and max at T2. **(B)** Trend of LDL/HDL-C ratio (normal-weight: *χ* ^2^ 7, p 0.030*; overweight/obese: *χ* ^2^ 1.5, p 0.473) and TG/HDL-C ratio (normal-weight: *X* ^2^ 1.6, p 0.449; overweight/obese: *χ* ^2^ 4.6, p 0.096) over time in the 2 groups (Friedman ANOVA test, * p<0.05). Dataare expessed as median, 25°-75° percentile and range (min-max).

In the total population, glucose metabolism got worse during GnRHas treatment; however, at nFH/FH, FGIR and HOMA index values were in the normal range. These values remained higher in group B than in group A throughout follow-up (**Figure 3A**).

Lipid metabolism did not significantly change over time in both the total population and group B patients (ratio of LDL-C to HDL-C: *χ* ^2^ = 4.2, *p* = 0.122; ratio of TGs to HDL-C: *χ* ^2^ = 0.75, *p* = 0.683; ratio of LDL-C to HDL-C: *χ* ^2^ = 1.5, *p* = 0.475; ratio of TGs to HDL-C: *χ* ^2^ = 4.6, *p* = 0.096) respectively. In group A only, the ratio of LDL-C to HDL-C transiently deteriorated during GnRHas treatment (ratio of LDL-C to HDL-C: *χ* ^2^ = 7, *p* = 0.030; ratio of TGs to HDL-C: *χ* ^2^ = 1.6, *p* = 0.449) (**Figure 3B**). No cases of diabetes, pre diabetes, or dyslipidemia were detected during and after GnRHas treatment.

## 4 Discussion

Childhood obesity remains one of the major global health problems (31). Recent Italian data confirm a mean prevalence of weight excess in female patients aged 3–17 years of 23.2%, a prevalence almost double that previously reported (32). As previously described, childhood obesity could be associated not only with cardiometabolic and psychosocial comorbidity and premature adult mortality but also with some endocrine complications, such as early or precocious pubertal onset. In our cohort, 42% of the enrolled girls were classified as being overweight or obese at diagnosis of ICPP. This prevalence is similar to previous reports, which reported a range from approximately 32% to 73% (33–39). This wide range could be influenced by geographical issues, including different genetic and epigenetic backgrounds, and different environmental settings. Furthermore, in our study ICPP manifested more precociously in overweight and obese girls than in normal-weight girls, which supports the etiopathogenic role of weight excess in precocious pubertal onset.

In our cohort, analyzing baseline data, overweight and obese girls seemed only apparently taller than normal-weight girls; in fact, H SDS was similar between groups when corrected according to BA. The absence of BA discrepancy between groups could be because ICPP advanced BA regardless of the patient’s initial BMI. In the same way, the initial picture of ICPP was clinically (as measured using Tanner’s breast stage) and biochemically similar in both groups. Therefore, we can postulate that overweight and obesity can act as a precocious trigger and, thus, anticipate but neither modify nor accelerate the etiopathogenic pathways of ICPP.

The longitudinal analyses of our data suggest a benefit of GnRHas in overweight and obese ICPP patients. It has been reported previously that obese children tend to lose their prepubertal growth advantage over time, mainly because of a decreased pubertal growth spurt (40). Prepubertal fast growth seems to be dependent on the increased bioavailability of IGF-1 as a result of hyperinsulinemia and on the skeletal growth-promoting effect of increased leptin levels on chondrocyte proliferation and maturation. In ICPP, the main goal of GnRHas treatment is to increase predicted adult height by preventing premature closure of the epiphyses (16). In our setting, nFH/FH SDS (as well as height gain) was comparable between normal-weight and overweight/obese girls. These data demonstrate that GnRHas reduces the advancement of bone maturation efficiently in both groups, supporting a similar continuous linear growth during treatment, and allowing a comparable growth spurt in all patients after its end. Recently, Lee et al. (41) documented the lowest growth gain from GnRHas treatment cessation to menarche (mean time lag 1.58 ± 0.53 years) in 10 girls obese at ICPP diagnosis compared with 13 overweight and 65 normal-weight girls. In our study, it was not possible to split the effect of overweight from obesity because of the small number of obese girls in our cohort. Therefore, we cannot conclude that our results are completely in contrast with Lee et al.’s (41) observations. Nevertheless, these authors considered the growth rate in the 3 months preceding menarche, whereas our study included a longer follow-up period; in girls, most of the growth spurt occurs before the menarche, but it typically continues afterwards (42).

Our longitudinal analyses also documented a significant increase in BMI SDS during GnRHas treatment in normal-weight girls, but not in overweight and obese girls. In addition, when nFH or FH was reached, BMI SDS returned near to pretreatment values in all ICPP girls (**Figure 2**). Our findings are in line with some previous data (36, 39, 43–45), but are discrepant from others (33, 37). No lifestyle intervention was carried out in any group; therefore, differences in BMI SDS pattern cannot be attributed to lifestyle. In the normal-weight group, our data support the hypothesis of a direct effect of GnRHas treatment in increasing adiposity despite the absence of an unfavorable anthropometric condition at ICCP diagnosis. The etiopathogenic hypotheses posit a temporary “menopausal effect” (46). On the other hand, the persistence of weight excess on GnRHas treatment in group B, even without further weight gain, reinforces this hypothesis and suggests that the peripheral hormonal effect of adiposity, mediated by aromatase, can also contribute to the precocious onset of menarche, despite therapy, in this group of patients in comparison with normal-weight girls (together with their earlier age at diagnosis) (47).

Only a few studies have analyzed the effects of GnRHas treatment on glucose and lipid metabolism in treated ICPP girls; however, these studies report discordant outcomes (34, 38, 48). Sørensen et al. (49) observed a significant worsening of insulin sensitivity and a significant increase in TGs, LDL-C levels and body fat percentage during 1 year of GnRHas treatment in 23 girls with ICPP. Chiavaroli et al. (50) demonstrated a higher HOMA IR value at final height in GnRHas-treated girls than in untreated girls. By contrast, Colmenares et al. (34) documented the persistence of normal lipid and glucose profiles during 3 years of follow-up among 43 patients with a diagnosis of ICPP or early puberty and 28 control participants. In 2015, an Israeli study published data on the risk of obesity, metabolic derangements, and cancer morbidities in a historical cohort of young women with a former diagnosis of childhood ICPP; using a national register, the authors demonstrated that in the women with a history of ICPP, whether treated or untreated, the percentage of hyperlipidemia, diabetes, and hypertension was relatively small, with no significant difference compared with their respective control participants, and between treated and untreated cohorts (43). One other study analyzed the metabolic effect of GnRHas treatment in an ICPP population based on pretreatment BMI; they demonstrated that, after 1 year of treatment, normal-weight patients showed an increase in BMI SDS, but not in HOMA-IR, whereas no changes were found in BMI SDS and HOMA-IR values in overweight and obese patients (48). To our knowledge, there are no more recent long-term metabolic data in the literature. In our cohort, glucose metabolism got worse during GnRHas treatment but was restored completely afterward. The slight increase in HOMA index value, occurring between T1 and T3, must be considered to be physiological because it also happens in healthy untreated girls. Moreover, the LDL-C-to-HDL-C ratio transiently deteriorated during GnRHas treatment in group A only; a trend that is not surprising. It is well known that levels of lipids fluctuate during childhood and adolescence and that the nadir of LDL-C is reached just during puberty (51–53). One other hypothesis speculates that GnRHas treatment acts with pituitary gonadotroph desensitization, and the subsequent gonadal steroid suppression produces a temporary “menopausal effect”. The levels of LDL-C peaked during the menopausal transition and the early postmenopausal stage, as was observed in our normal-weight cohort. Some authors suppose that, in ICPP girls treated with GnRHas, physiological estrogen production was suppressed by therapy, leading to estrogen depletion and an excessive androgen exposition with a subsequent clinical and/or biochemical relative hyperandrogenism. All these changes could be responsible for the accumulation of body fat over lean body mass and the subsequent metabolic adverse consequences (49, 54). Furthermore, increased adiposity in postmenopausal women is significantly associated with hyperinsulinemia, which suggests that insulin resistance may be responsible for the development of the key feature of postmenopausal dyslipidemia and metabolic syndrome (55).

Limitations of our study include its retrospective design and the absence of a control (i.e., untreated ICPP) group. Furthermore, because of its retrospective design, our study did not assess other parameters of adiposity, such as waist circumference, body composition and other metabolic risk factors (e.g., familial cardiovascular risk, dietary habit, sedentary lifestyle, and socioeconomic status), and other metabolic parameters (e.g., inflammation markers or adipokines). These issues could be solved by carrying out an appropriate prospective phase of the study. On the other hand, the strengths of the study were that all of the cases were followed up in a single center, which limited variation in anthropometric, biochemical, instrumental, and therapeutic applied methods, and that the size of our sample was similar and not inferior to other studies.

In conclusion, our long-term anthropometric outcomes confirm the efficacy of GnRHas on growth and suggest that overweight and obese girls at the point of diagnosis of ICPP benefit from GnRHas treatment similarly to normal-weight peers. However, the observed transient impairment of metabolic parameters during treatment should stimulate clinicians to encourage an adequate and healthy lifestyle in all ICPP girls treated with GnRHas, regardless of their pretreatment BMI.

## Data availability statement

The raw data supporting the conclusions of this article will be made available by the authors, without undue reservation.

## Ethics statement

The studies involving human participants were reviewed and approved by AOU Modena. Written informed consent to participate in this study was provided by the participant’s legal guardian/next of kin.

## Author contributions

LI and PB conceptualized the study, analyzed and interpreted the data, and wrote the manuscript. LV and MS contributed to data collection and fulfilled the database. LL, BP, and SM helped to analyze the data and performed a critical revision of content. All the authors approved the final version, and all agreed to be accountable for all aspects of the work in ensuring that questions related to the accuracy or integrity of any part of the work are appropriately investigated and resolved.

## Conflict of interest

The authors declare that the research was conducted in the absence of any commercial or financial relationships that could be construed as a potential conflict of interest.

## Publisher’s note

All claims expressed in this article are solely those of the authors and do not necessarily represent those of their affiliated organizations, or those of the publisher, the editors and the reviewers. Any product that may be evaluated in this article, or claim that may be made by its manufacturer, is not guaranteed or endorsed by the publisher.
